# A Field Procedure for the Assessment of the Centring Uncertainty of Geodetic and Surveying Instruments

**DOI:** 10.3390/s18103187

**Published:** 2018-09-20

**Authors:** José L. García-Balboa, Antonio M. Ruiz-Armenteros, José Rodríguez-Avi, Juan F. Reinoso-Gordo, Juan Robledillo-Román

**Affiliations:** 1 Departamento de Ingeniería Cartográfica, Geodésica y Fotogrametría, Universidad de Jaén, 23071 Jaén, Spain; amruiz@ujaen.es; 2 Centro de Estudios Avanzados en Ciencias de la Tierra (CEACTierra), Universidad de Jaén, 23071 Jaén, Spain; 3 Departamento de Estadística e Investigación Operativa, Universidad de Jaén, 23071 Jaén, Spain; jravi@ujaen.es; 4 Departamento de Expresión Gráfica Arquitectónica y en la Ingeniería, Universidad de Granada, 18071 Granada, Spain; jreinoso@ugr.es; 5 Independent Researcher, 18011 Granada, Spain; jrobledilloroman@gmail.com

**Keywords:** centring error, uncertainty, plummet, tribrach, total station, GNSS, TLS

## Abstract

The uncertainty evaluation of survey measurements is a daily and essential task in any surveying work. The result of a measurement is, in fact, only complete when accompanied by a statement of its uncertainty. Miscentring, or centring error, is one of the sources of uncertainty in every basic survey measurement which may have a great effect on horizontal angle measurement for short distances. In the literature, different terms and values are considered to refer to this source of uncertainty. Standard ISO 17123 provides different procedures for assessing the measurement uncertainty of geodetic and surveying instruments, with the aim of checking their suitability for the intending and immediate task in field conditions. ISO 17123 is aware of the importance of uncertainty in the instrument centring, but it does not propose any standardised procedure for its assessment. In this study, we propose such a procedure following a Type A evaluation (through the statistical analysis of series of observations), avoiding using values from Type B evaluations (from manufacturer’s specifications, handbooks, personal experiences, etc.) that could be unsuitable for the conditions of the task. Uncertainty can be individualised for a particular instrument (which includes the plummet type), ground mark, operator, and other factors on which the results could be dependent. The testing methodology includes a configuration of the test field, measurements, and calculation, following the structure of each part of the standard ISO 17123. An experimental application is included with two different total stations, which also includes a statistical analysis of the results.

## 1. Introduction

The uncertainty evaluation of survey measurements is a daily and essential task in any surveying work. The result of a measurement is, in fact, only complete when accompanied by a statement of its uncertainty [1]. If a required accuracy needs to be satisfied, the uncertainty evaluation of the measurements allows us to decide about the most suitable instruments and measurement procedure. An example for the setting out of a point can be found in [2].

According to the Guide to the Expression of Uncertainty in Measurement (GUM) [1], the uncertainty of any quantity derived from other quantities can be evaluated by the law of propagation of uncertainty. In this way, the uncertainties of the basic survey measurements (distances, horizontal directions, zenith angles, etc.) are propagated to evaluate the uncertainties of derived measurements (coordinates, surfaces, volumes, etc.) in which they are used. Only then will the results of such derived measurements be complete. Therefore, the first stage is to carry out the uncertainty evaluation of the basic survey measurements. This will determine the reliability of subsequent results of uncertainty for derived quantities. 

There are a great number of sources of uncertainty in every basic survey measurement. In practice, only some of them are significant when evaluating the uncertainty of a measurement (see [3]). One of them is miscentring, or the centring error, over the survey mark when setting up the measurement instrument (offset from the true station centre). In [4], it is defined as the plane projection of the deviation of the instrument’s measuring centre from the ground point including the uncertainty due to the levelling of the tribrach. The accuracy of measurements from a total station, a Global Navigation Satellite System (GNSS) receiver or a terrestrial laser scanner (TLS) is still highly influenced by this error, despite the evolution in instrument manufacturing which has removed different errors that occurred in the past. 

The uncertainty of the centring of an instrument $s_{CI}$ relates to the horizontal positional uncertainty of the bottom plate of a tribrach of a tripod-mounted instrument above a ground survey mark, or of a pillar-mounted instrument around the centre mark of the pillar. Mainly in horizontal angle measurement, this source of uncertainty may have a great effect for short distances, which emphasises the need to set up the instrument carefully. In [5], different charts are presented with the contribution to the uncertainty of a horizontal angle due to the uncertainty in the instrument centring. As an example, a centring error of 1 mm causes an error of 40^cc^ (≈ 13′′) in a horizontal angle of 150 grad (= 135°) in sights of 30 m. Because of its importance in the quality of a measure, different authors continue investigating the assessment of this uncertainty [4,6,7], the minimization of its influence [8] or its influence in error models [5,9]. Certain brands also continue improving the centring accuracy, for example the Trimble SX10, which takes an image of the ground mark [10].

This uncertainty in instrument centring $s_{CI}$ depends on: (i) the state of the plummet system (mainly optical or laser); (ii) the quality of the tripod; and (iii) the skill of the surveyor [11]. Many problems involved with instrument centring stem from a careless attitude on the part of the surveyor and especially a failure to test the plummet and ensure that the instrument is carefully levelled [5]. Some other parameters that influence $s_{CI}$ are the quality of lighting, the resolution of the observer’s eye, the plummet zoom, and the height of the instrument over the ground target [4]. Different authors quantify this source of uncertainty from 0.5 to 5 mm depending on the plumbing system (plumb bob, centring rod, optical plummet or laser plummet; see Table 1). On the other hand, for a forced (constrained) centring system, the uncertainty is quantified below 0.3 mm. In the literature, different terms are used to refer to this source of uncertainty, mainly accuracy, standard deviation, or maximum error, but they are not equivalent terms (see [12]). According to [1], each component that contributes to a measurement uncertainty should be represented by a standard deviation termed “standard uncertainty”. For the sake of brevity, in this paper, we use the term “uncertainty” to refer to “standard uncertainty”. Standard uncertainty is also denoted by *s*, independently of whether it comes from an empirical value.

Standard ISO 17123 provides different procedures for assessing the measurement uncertainty of geodetic and surveying instruments, with the aim of checking their suitability for use in field conditions. Manufacturers usually follow this standard to indicate the uncertainty of measurement of their instruments. Nevertheless, it is important to note that they perform the procedures in testing laboratories under optimal conditions, thus obtaining uncertainty values lower than those obtained in field conditions. The proposed tests must not be confused with calibration procedures, which aim to introduce corrections to the measurement results due to the presence of systematic measurement errors and must always be performed in testing laboratories.

To date, ISO 17123 is structured in eight parts: Part 1 [21] is dedicated to theory, while Parts 2–8 focus on levels (measurand is height difference in 1 km double-run levelling), theodolites (horizontal direction observed in both faces of the telescope), electro-optical distance meters (single measured distance), total stations (x, y coordinates on both faces of the telescope), rotating lasers (height difference between the instrument and a levelling staff), optical plumbing instruments (horizontal displacement of a point transferred over the plumbing height), and GNSS (horizontal position and height in real-time kinematic), respectively. Part 9 is under development and will be dedicated to TLS [22]. The aim of ISO 17123 is not to assess each of the sources of measurement uncertainty, but the uncertainty of the final result (a black-box approach), which is what worries a typical user in a practical context. A Type A evaluation of uncertainty (statistical analysis of a series of observations) [1] is proposed for each measurand.

ISO 17123 is aware of the importance of uncertainty in instrument centring, but it does not propose any standardised procedure for its assessment. Part 3 (theodolites) declares that “special care shall be taken when centring above the ground point”. Part 8 (GNSS in RTK) requires a $s_{CI}$ of 1 mm to perform the test. Part 7 (optical plumbing instruments) could apparently be related to the assessment of instrument centring, but it clarifies that the proposed test is not applicable to optical plummets as a device in tribrachs or in surveying instruments. Furthermore, Part 5 (total stations) includes a list of sources of uncertainty not to be evaluated individually. The centring of a total station is included in the list since it has been considered when evaluating uncertainty in distances, vertical angles, and horizontal angles. 

In this paper we develop a standardised procedure for the assessment of the uncertainty in instrument centring. In this way, any user of a geodetic or surveying instrument could perform a Type A evaluation avoiding using values from Type B evaluations (from manufacturer’s specifications, handbooks, personal experiences, etc.) that could be unsuitable for the conditions of the intended task. The uncertainty could be individualised for a particular instrument (which includes the plummet type), ground mark, operator, and other factors on which the results could be dependent. The procedure is designed considering the general criteria of the standard ISO 17123, and therefore developed for in situ applications without the need for special ancillary equipment. The testing methodology is shown in Section 2, including the configuration of the test field, measurements and calculation. An experimental application is summarised in Section 3. Finally, the conclusions are included in Section 4.

The angular units used in this paper are gradian (grad) and centesimal second (^cc^), since they are used by the surveying community in different countries. To assist the reader the corresponding values in degrees and seconds of arc are given (in brackets) for some results. The following relationships hold: 400 grad = 360°; 1 grad = 10,000^cc^; 1^cc^ = 0.0001 grad; 1 grad = 0.9°; 1′′ ≈ 3^cc^ (3.086^cc^); and 1^cc^ ≈ 0.3′′ (0.324′′).

## 2. Testing Methodology

**Figure 1:**
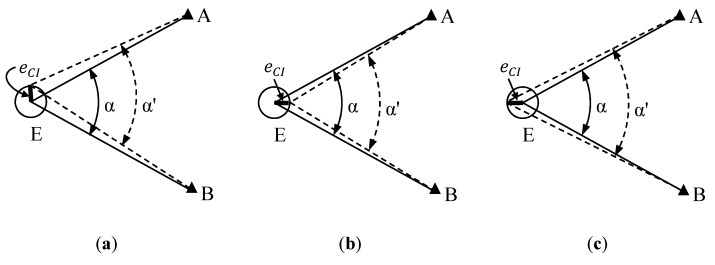
Effect of error in the instrument centring $e_{CI}$ on a measured horizontal angle *α*. It does not affect the angle in Case (**a**), but the effect is maximum in Cases (**b**) and (**c**). Erroneous lines of sight and angle indicated by dashed lines.

The main objective of the proposed testing procedure is to assess the uncertainty in the centring of an instrument. By instrument, we mean any geodetic or surveying equipment which needs to be set up exactly over a ground mark, usually with the assistance of optical or laser plummets associated to tribrachs. Therefore, theodolites, total stations, GNSS receivers or TLS could be considered. As presented in Section 1, the general criteria of standard ISO 17123 imply not requiring the use of special ancillary equipment. The great influence of the centring error in the horizontal angle measurement has been demonstrated. A testing procedure based on both considerations is proposed. The foundation of the method lies in the setting up of two stable targets at short distances from the instrument so that a small displacement in its position (due to the centring error) leads to a significant variation of the horizontal angle between both targets. A similar strategy can be found in [7], in this case to check the influence of the imperfection of construction of the tribrach. That approach is exactly the opposite of the configuration of the test field in standard ISO 17123-3, which locates the targets 100–250 m away to minimise the influence of the centring error. Figure 1 illustrates a horizontal angle *α* measured from Station E to Targets A and B. An error in the instrument centring $e_{CI}$ does not affect angle *α* when the instrument is located in the position shown in Figure 1a, that is on the circumference containing E, A, and B (*α* ≅ *α*’). In contrast, the effect is maximum when the instrument is positioned along the angle bisector (Figure 1b,c) (*α* ≠ *α*’) [3]. 

To find a recommended distance between the instrument and the targets, the contribution to the uncertainty of a horizontal angle due to the uncertainty in instrument centring $s_{CI}$, denoted by $s_{\alpha_{CI}}$, has to be considered. It can be evaluated as (see page 185, Equation (7-7) in [19], page 177, Equation (6.9a) in [23], page 123, Equation (7.21) in [3], among others):

(1) $$s_{\alpha_{CI}}=\frac{\sqrt{{\left(D_{E}^{A}\right)}^{2}+{\left(D_{E}^{B}\right)}^{2}-2D_{E}^{A}D_{E}^{B}cos\alpha }}{D_{E}^{A}D_{E}^{B}}s_{CI}=\frac{D_{A}^{B}}{D_{E}^{A}D_{E}^{B}}s_{CI}=\frac{D_{3}}{D_{1}D_{2}}s_{CI}\;$$

Assuming that the distances between the instrument and the targets are equal ($D_{1}=D_{2}=D$) and α=100 grad (a right angle), Equation (1) can be simplified as:

(2) $$\;s_{\alpha_{CI}}=\frac{D\sqrt{2}}{D^{2}}s_{CI}=\frac{\sqrt{2}}{D}s_{CI}\;$$

Table 2 presents some results from the application of Equation (2). Assuming three different values for $s_{CI}$ (0.33 mm, 0.5 mm and 1 mm) and five values for D (5, 10, 15, 20 and 25 m), we can explore the values obtained for $s_{\alpha_{CI}}$.

The value of $s_{\alpha_{CI}}$ should be significantly larger than other sources of uncertainty in the horizontal angle. If we take the smallest value for $s_{CI}$ (0.33 mm) and the largest value for D (25 m), it results in the lowest value of $s_{\alpha_{CI}}$ (12^cc^ ≈ 4′′). In this case, $s_{\alpha_{CI}}$ is not significantly larger, because usual values for $s_{ISO-THEO-Hz}$ (standard uncertainty in a horizontal direction observed in both faces of the telescope (ISO 17123-3)) in standard total stations are in the range of 10–20^cc^ (≈ 3–6′′). If we take the largest value for $s_{CI}$ (1 mm) and the smallest value for D (5 m), it results in the largest value of $s_{\alpha_{CI}}$ (180^cc^ ≈ 60′′). In this case, it can be assumed that $s_{\alpha_{CI}}$ is significantly larger and therefore the uncertainty in the horizontal angle $s_{\alpha }$ can be approximated to $s_{\alpha_{CI}}$. It is supposed that, as stated in the different procedures of ISO 17123, the instrument and ancillary equipment is known and acceptable states of permanent adjustment according to the methods specified in the manufacturer’s handbook, and used with tripods as recommended by the manufacturer.

It is obviously preferable for $s_{ISO-THEO-Hz}$ to be as small as possible, but a relation with $s_{\alpha_{CI}}$ should be stated. We assume that $s_{\alpha }=\sqrt{s_{\alpha_{ISO}}^{2}+s_{\alpha_{CI}}^{2}}≅s_{\alpha_{CI}}$ if the contribution to the angle uncertainty from the instrument $s_{\alpha_{ISO}}=s_{ISO-THEO-Hz}\sqrt{2}$ is 3 (or more) times smaller (better) than $s_{\alpha_{CI}}$. For example, if a total station with $s_{ISO-THEO-Hz}=15^{\mathrm{cc}}$ (≈ 5′′) is considered, $s_{\alpha_{CI}}$ should be $15\sqrt{2}\times 3=63.6^{\mathrm{cc}}$ (≈ 21′′) (or more). From Equation (2), we can find that, if $s_{CI}=0.33$ mm, then D needs to be 5 m or less; if $s_{CI}=0.5$ mm, then D needs to be 7 m or less; and, if $s_{CI}=1$ mm, then D needs to be 15 m or less. In all these three cases, $s_{\alpha }=\sqrt{s_{\alpha_{ISO}}^{2}+s_{\alpha_{CI}}^{2}}=\sqrt{{\left(15\sqrt{2}\right)}^{2}+63.6^{2}}=67.0≅s_{\alpha_{CI}}=63.6^{\mathrm{cc}}$. 

Not only one but two angles, *α* and *β*, should be considered given that the $e_{CI}$ is two-dimensional. As can be seen in Figure 1, *α* is sensitive to the component of $e_{CI}$ which is parallel to its bisector; therefore the bisectors of *α* and *β* should be perpendicular.

**Figure 2:**
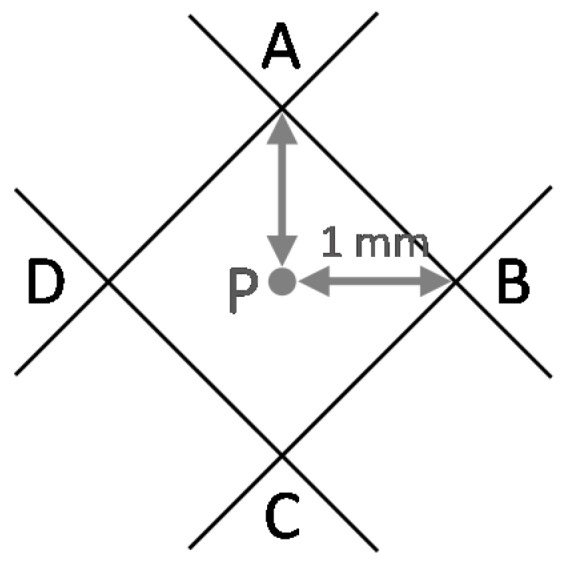
Ground mark employed for the preliminary test. Four positions (A–D) were established from a centre point P. Each position is separated from P with a $e_{CI}$ = 1 mm. Elements in grey are included here for explanation but not printed in the real mark.

**Figure 3:**
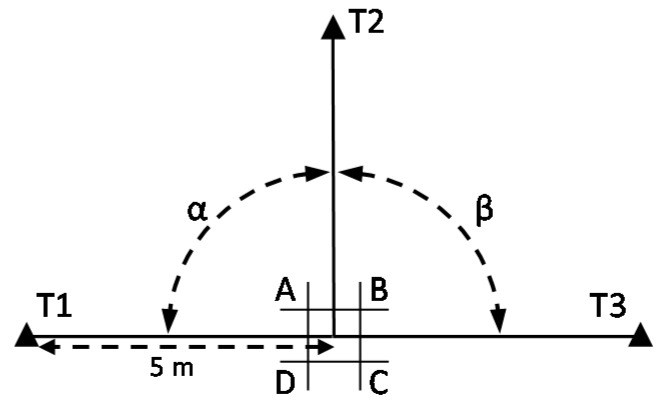
Design of the test field for the preliminary test (ground mark not to scale). Angle *α* is measured from Positions A and C. Angle *β* is measured from Positions B and D.

**Figure 4:**
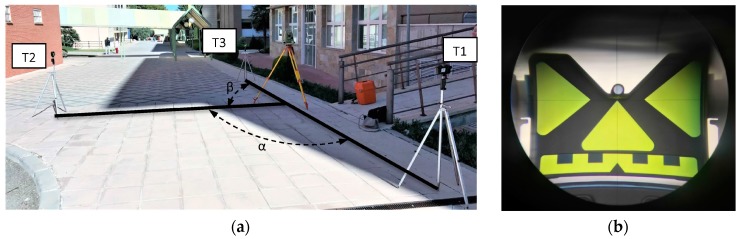
(**a**) Setup for preliminary test; (**b**) A pointing to the target plate, model Leica GRZ3.

A preliminary test with real data was performed to determine whether $e_{CI}$ can be estimated from the measurement of *α* and *β*. Small predefined displacements of the instrument were introduced, which should be clearly detected. An adhesive paper ground mark with four positions (A–D) was designed (Figure 2), each one displaced 1 mm from a centre point P. Three stable target plates (T1–T3) were set up, considering two conditions: they form two adjacent right angles and D = 5 m from P (Figure 3 and Figure 4a). These conditions can be approximated; accurate conditions are not required. The instrument was set up over each of the four positions and *α* and *β* were measured (both faces of the telescope). A Leica TC-1800 total station was used, with $s_{ISO-THEO-Hz}$ = 3^cc^ (manufacturer specifications), optical plummet in tribrach, and dual-axis compensator. For the selection of the target plate, we performed a simple analysis of repeatability (standard deviation from 20 measures) in pointing to the target plate of a Leica GPR111 standard circular prism and to a Leica GRZ3 target plate without prism (Figure 4b), with better results in the latter (3^cc^ ≈ 1′′). 

In Table 2, it can be seen that, when $s_{CI}$ = 1 mm and D = 5 m, a variation in the horizontal angles of 180^cc^ (≈ 60′′) is expected. Therefore, twice this value, 360^cc^ (≈ 120′′), is expected when comparing the value of *α* measured from A and C (Δ*α* = *α*_A_ − *α*_C_) and when comparing the value of *β* measured from B and D (Δ*β* = *β*_B_ − *β*_D_). In Table 3, we present the results, which are Δ*α* = 387^cc^ and Δ*β* = 401^cc^, which represent a displacement of 2.2 mm, close to the predefined value of 2.0 mm. These results confirm the theoretical data and indicate that this configuration of the test field is sensitive to instrument centring. Therefore, we have confidence to take the next step and configure the testing methodology for the assessment of $s_{CI}$. 

If we fix the conditions of the preliminary test (value of D = 5 m and observations in both face positions of the telescope), we can relate the uncertainty in instrument centring $s_{CI}$ and the uncertainty in the horizontal direction $s_{ISO-THEO-Hz}$. It should be remembered that we assume that $s_{\alpha_{ISO}}=s_{ISO-THEO-Hz}\sqrt{2}$ ≤ $\frac{s_{\alpha_{CI}}}{3}$, therefore $s_{ISO-THEO-Hz}$ ≤ $\frac{s_{CI}}{3\cdot D}$. Table 4 shows the maximum value (it could be smaller, i.e., better) of $s_{ISO-THEO-Hz}$ required for the instrument to assess different expected values of $s_{CI}$ in a range between 0.1 and 1 mm. As an example, only when using very accurate total stations ($s_{ISO-THEO-Hz}$ ≤ 4^cc^) could a $s_{CI}$ = 0.1 mm be assessed; however, if a value of $s_{CI}$ = 0.5 mm is considered, a standard total station ($s_{ISO-THEO-Hz}$ ≤ 21^cc^) can be used. Table 5 shows the minimum value (it could be larger, i.e., worse) of $s_{CI}$ which can be assessed for different values of $s_{ISO-THEO-Hz}$ in a range between 5^cc^ (≈ 1.6′′) and 30^cc^ (≈ 10′′). As an example, if we have an instrument with $s_{ISO-THEO-Hz}$ = 10^cc^ (≈ 3′′), an instrument centring of $s_{CI}$ ≥ 0.24 mm could be assessed.

The following subsections provide details of the proposed testing methodology. They are organised in a similar way to ISO 17123: configuration of the test field, measurements, and calculation.

In contrast to previous studies (e.g., [4,6,8]), this proposal can be applied to an instrument with any type of plummet (laser or optical) and does not need any additional equipment. It can be applied directly to theodolites and total stations. In the case of GNSS receivers, it could be applied if their tribrachs are interchangeable and they incorporate the plummet; a total station or theodolite should be mounted over this tribrach to perform the test. It could also be adapted for TLS in a similar way as ISO 17123-3 is adapted in [24] for horizontal directions.

### 2.1. Configuration of the Test Field

In line with the structure of each part of the standard ISO 17123, a configuration of the test field should be performed to standardise it. We follow the same drafting style of ISO 17123-3 to define the configuration that has already been used in the preliminary test.

**Figure 5:**
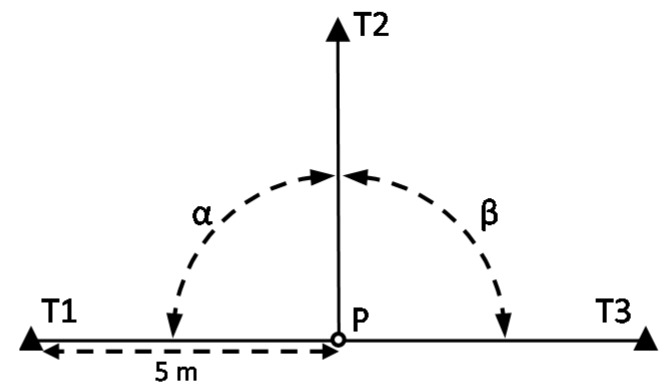
Configuration of the test field.

An instrument, ground mark, and operator should be chosen for the test. The test area is that which surrounds the ground mark at position P. Three fixed targets (T1–T3) shall be set up located approximately on the same horizontal plane as the instrument, 5 m away, and situated forming two adjacent right angles (*α* and *β*) (see Figure 5). Targets shall be used which can be observed unmistakably, preferably target plates. They shall be stable during the test measurements. The conditions of distance to the targets and right angles can be approximated; accurate conditions are not required.

Table 4 can be used to know if the instrument is accurate enough (more specifically, its value of $s_{ISO-THEO-Hz}$) to assess an expected uncertainty in instrument centring. Similarly, Table 5 can be used to know the smallest value of uncertainty in instrument centring that can be assessed with that instrument.

### 2.2. Measurements

As indicated in [25], standard ISO 17123 defines two test types that vary in complexity: the simplified and full test procedures. The first is faster, based on small sample of measurement, with limited significance of the results and only useful as an indication of the order of the measure of the achievable precision (statistical tests are not proposed). The latter requires a larger sample size and the computed standard deviation is more significant for the uncertainty assessment. The sample size for our proposal is discussed below.

Consecutive *n* setups of the instrument should be carried out over the ground mark. Special care should be taken when centring. In each setup, a series of measurements shall be taken, following a measurement procedure similar to that of ISO 17123-3. The three targets shall be observed in each set in Face Position I of the telescope in clockwise sequence, and in Face Position II of the telescope in anticlockwise sequence, noting the value of the six horizontal directions. The pointing error should be minimised by bisecting with the cross-hair reticle. 

After each setup, the tribrach shall be turned approximately 133 grad (120°). This way, the independence between consecutive setups is guaranteed and a systematic error introduced by plummets integrated in tribrachs could be detected and also included in the uncertainty result (as indicated by [1,12]). In addition, systematic errors in the centring due to the mark shape or illumination (and shadows) would be included in the result. Nevertheless, this condition could slow down the procedure, making it less practical. As an alternative, the *n* setups (series) could be divided into *n*/3 groups, each one with the same tribrach orientation. To maintain the independence in the centring procedure, the instrument shall be off-centred between consecutive setups in the same group.

The instrument height can influence the results. A comfortable height, mostly used by the operator–instrument pair, should be chosen for every setup. Another alternative is to introduce variability in the height to obtain uncertainty results for a broader range of heights. The choice should be made depending on the intended objective.

**Figure 6:**
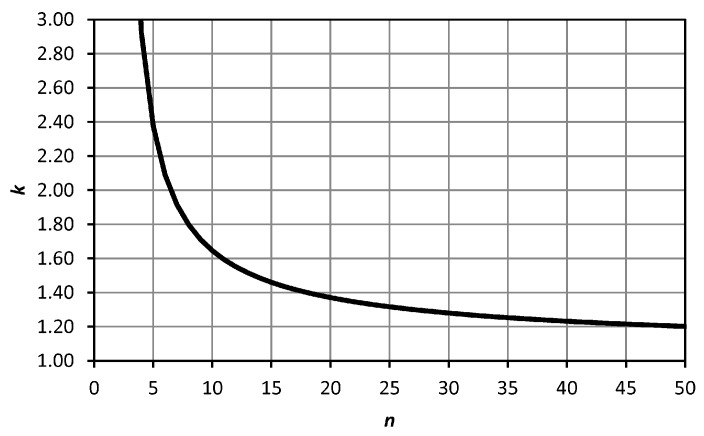
Confidence interval for the standard deviation. The line represents how many times the upper limit (*k*) is larger than *s* (5% level of significance for a one-tailed test) for each sample size *n*.

The sample size *n* is a major issue. In the full test procedure, it should be small enough to speed up the operation and large enough to obtain significant results. The confidence interval for the standard deviation can be considered. If the data are normally distributed, the expression $\frac{\left(n-1\right)s^{2}}{\sigma^{2}}$ has a chi-square distribution with (n−1) degrees of freedom, where *σ* is the population standard deviation and *s* the sample standard deviation. Taking the upper limit of the confidence interval and a 5% level of significance for a one-tailed test, Figure 6 shows how many times (*k*) this limit is greater than *s* for each sample size *n*.

In Figure 6, *k* is below 2.0 for *n* = 7, below 1.5 for *n =* 14, below 1.4 for *n =* 18, below 1.3 for *n =* 27 and below 1.2 for *n =* 49. We suggest for the full test procedure a value of *n =* 24 (*k =* 1.33) as a compromise between significance and practicality. It is estimated that the field work could be carried out in half a working day (about 4–5 h). In any case, *n* should be a multiple of 3, due to the turns of the tribrach. For the faster simplified test procedure, a value of *n =* 6 could be considered, remembering that it is only useful as an indication of the order of the measure of the precision achievable.

### 2.3. Calculation

First, the mean values of the readings in both Face Positions I and II of the telescope are calculated:

(3) $$\;x_{i,j}=\frac{x_{i,j,I}+x_{i,j,\;II}}{2},\;$$

where *i* = 1, …, *n* and *j* = 1, 2, 3, corresponding to points to T1–T3.

Next, the horizontal angles *α* and *β* are obtained for each series *i*:

(4) $$\alpha_{i}=x_{i,2}-x_{i,1},\;\beta_{j}=x_{i,3}-x_{i,2}.\;$$

Then, the standard deviation values of *α* and *β* are computed:

(5) $$\;s_{\alpha }=\frac{\sum_{j=1}^{n}\left(\alpha_{j}-\bar{\alpha }\right)}{n-1},\;s_{\beta }=\frac{\sum_{j=1}^{n}\left(\beta_{j}-\bar{\beta }\right)}{n-1}\;$$

where $\bar{\alpha }$ and $\bar{\beta }$ are the mean values of *α* and *β*, respectively.

The two-dimensional components of $s_{CI}$ are:

(6) $$\;s_{CI-x}=s_{\alpha }\frac{D}{\sqrt{2}}\;;\;s_{CI-y}=s_{\beta }\frac{D}{\sqrt{2}}\;\;$$

Finally, the uncertainty in the instrument centring is:

(7) $$\;s_{CI}=\frac{s_{CIx}+s_{CIy}}{2}\;$$

which is an approximation of the circular standard deviation, valid when the quotient *s_min_*/*s_max_* is between 1.0 and 0.6 [26].

## 3. Experimental Application

The proposed testing methodology described in Section 2 was applied with two different instruments: a Leica TS-06 total station (TS1), with $s_{ISO-THEO-Hz}$ = 15^cc^ (≈ 5′′) (manufacturer specifications) and laser plummet and a Leica TC-1800 total station (TS2), with $s_{ISO-THEO-Hz}$ = 3^cc^ (≈ 1′′) (manufacturer specifications) and optical plummet in tribrach. In both cases, these remain the same: operator, tripod, target plates (Figure 4b), and configuration of the test field (Figure 5).

**Figure 7:**
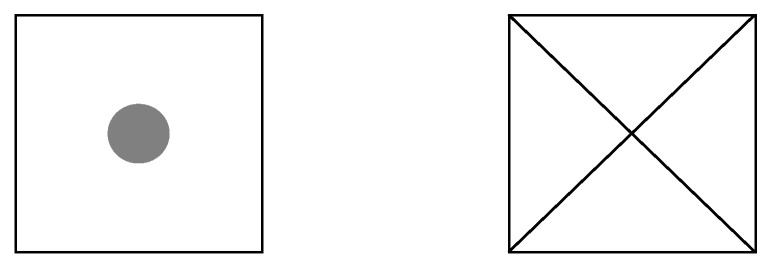
Ground marks employed for the experimental application (real size): (**left**) M1 is of a circular-type with a diameter of 5 mm; and (**right**) M2 is a cross-type with 0.5 points (about 0.2 mm) width.

For a deeper analysis of the methodology, the number of series *n* was increased over the one proposed in Section 2 (24 setups). In addition, different variables which could affect the results were introduced: two types of ground mark (M1 and M2) (Figure 7) and different instrument heights. For each mark and height, the three turns of the tribrach were considered and three setups were performed in each turn (see Table 6). The number of series is *n*_1_ = 54 for TS1 and *n*_2_ = 45 for TS2.

**Figure 8:**
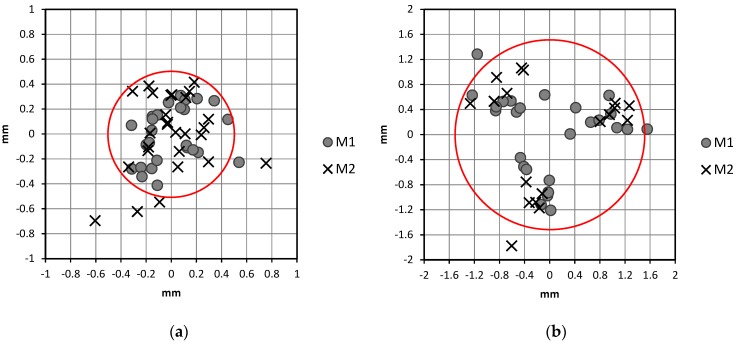
(**a**) Centring errors from setups performed with TS1 (laser plummet); and (**b**) centring errors from setups performed with TS2 (optical plummet). M1 and M2 are the ground marks in Figure 7.

Results from the *n*_1_ series of TS1 are presented in Figure 8a. Each point in the chart represents the centring error of each setup. Previously, each pair *j* of residuals $\alpha_{j}-\bar{\alpha }$ and $\beta_{j}-\bar{\beta }$ from Equation (5) was converted into lineal values $e_{CI-X_{j}}$ and $e_{CI-Y_{j}}$ (the two components of the centring error) to be plotted in the chart. A different symbol was considered for each type of ground mark. In 91.3% of setups, the centring error was less than 0.5 mm (red circle in the figure). From Equation (6), the two-dimensional components of $s_{CI}$ were $s_{CI-x}$ = 0.24 mm and $s_{CI-y}$ = 0.27 mm. Finally, from Equation (7), the uncertainty in the instrument centring was $s_{CI}$ = 0.25 mm. This is quite a good result, which confirms that special care has been taken by the operator and that the instrument is in good condition. In addition, it is important to note that it is better than that indicated by the manufacturer in [17] (see Table 1). An expanded uncertainty $U_{CI}$ can be obtained by applying different coverage factors (*k*), as suggested by [1]. Factors from a circular standard distribution can be obtained from [26]. For a probability of 90%, we can obtain $U_{CI-90}=k_{90}U_{CI}=2.1460\times 0.25=0.54$ mm and, for 99.8%, we can obtain $U_{CI-99.8}=k_{99.8}U_{CI}=3.5\times 0.25=0.89$ mm.

Similarly, results from the *n*_2_ series of TS2 are presented in Figure 8b. In 94.7% of setups, the centring error was less than 1.5 mm (red circle in the figure). The two-dimensional components of $s_{CI}$ were $s_{CI-x}$ = 0.77 mm and $s_{CI-y}$ = 0.74 mm. The uncertainty in the instrument centring was $s_{CI}$ = 0.76 mm. This result is quite different from that obtained from TS1. The uncertainty in the instrument centring was three times better (lower) in TS1 than in TS2. Moreover, the optical plummet of TS2 seems not to be in a good state. A systematic error introduced by the plummet system can be inferred in Figure 8b. Points in the chart seem to be separated into three groups, one for each turn of the tribrach. An estimation of the systematic error can be obtained if the mean is computed for each group, obtaining a result of 1.0 mm in the three turns. This is a considerable value and therefore the plummet system should be adjusted. In the meantime, this systematic error is included in the uncertainty result. 

### Statistical Analysis

A deeper analysis of the results is included in this section. Different tests were applied for the two components of the centring error $e_{CI-X}$ and $e_{CI-Y}$ (Figure 8a,b). They were performed using SPSS software, version 22.0 [27]. To make a final decision on each test, the *p*-values obtained have to be compared with the previously desired Type I error level *α* (usually 5% or 1%). The following assumptions were checked.

- Relation between the two components: In this case, an independence test on the Pearson correlation coefficient (*r* = 0.020 and *p*-value = 0.843) and also the Spearman rank correlation coefficients (*r_s_* = 0.0088 and *p*-value = 0.941) were performed (see, for example, [28]). This implies that both components are independent. Therefore, subsequent tests were carried out separately for each component.
- Normality of errors: In this case, separate tests were performed for each component ($e_{CI-X}$ and $e_{CI-Y}$) and each instrument (TS1 and TS2) with programs written in the R language [29]. Two tests were selected: Shapiro-Wilks [30] and Jarque-Bera [31]. Since the sample sizes were less than 5000 points, the robust version of the Jarque-Bera [32] was employed. Results are shown in Table 7. The results are not conclusive with a high evidence of non-normality. From Shapiro-Wilks tests, the assumption of normality could be rejected for a significance level of 0.05 in any case, but not for a level of 0.01. From Jarque-Bera tests, the assumption of normality was clearer, with *p*-values over 0.05, with the exception of component $e_{CI-X}$ of TS1. It is interesting to remember that ISO 17123-8 [33] (p. 10) assumes normality in the error centring.
- Outliers: We considered an outlier as a data point whose absolute value is greater than three times the standard deviation of the sample. Once the sample was standardised (for each component and each instrument), all points had a standardised absolute value lower than 3.
- Equality of variances: To contrast the equality of variances, we calculated the Levene’s test [34] of homogeneity of variances for each component and instrument. These comparisons were carried out for groupings from three different variables: type of ground mark (two groups, M1 and M2), instrument height (four groups, identified in Table 6), and tribrach turn (three groups). Results are shown in Table 8. They show that the centring error was not affected by these variables. Only in the case of instrument height for TS2, where the *p*-value is 0.045, could we find some influence in the variance of $e_{CI-Y}$ due to a significative difference between the highest and lowest height. Finally, we have proven that the variance was not homogeneous between instruments TS1 and TS2 from a global Levene’s test (Table 9) for two groups (two instruments).
- Homogeneity of means: This test was carried out to compare the tribrach turn in each instrument separately. Once we proved that the variances were homogeneous, an analysis of variance (ANOVA) was performed to compare the equality of error means [35]. The results (Table 10) imply that differences (bias) exist in instrument TS2 because the means of each position differ significantly.

**Figure 9:**
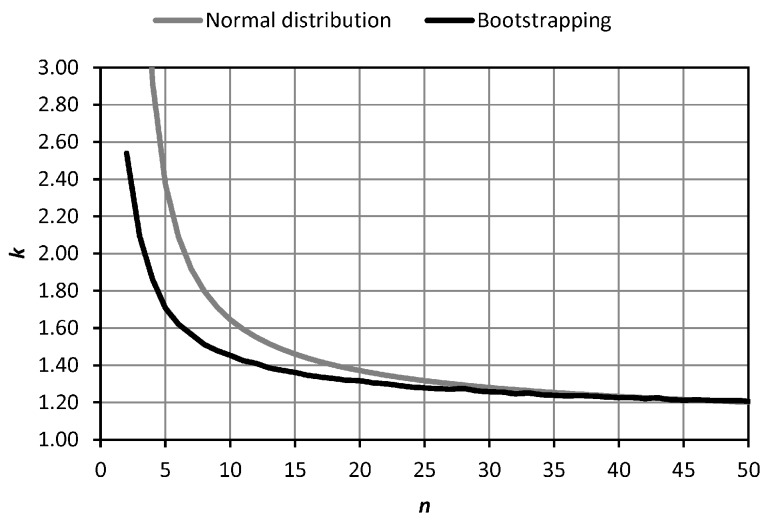
Comparison between the upper limit of the confidence interval (5% level of significance for a one-tailed test) for *s* (normal distribution, Figure 6) and the quantile of 95% of *s* (bootstrapping on centring error of TS1). Lines represent how many times (*k*) they are larger than *s* for each sample size *n*.

Additionally, a bootstrapping procedure [36] was developed for the centring error of TS1. All computations were performed using programs written in the R language [29]. Sampling sizes from 2 to 50 were chosen. For each sampling size, 10,000 samples were taken with replacement. For each sample, the standard deviation *s* was calculated. Finally, the quantile of 95% of the 10,000 standard deviation was obtained for each sample size. The objective was to compare the results with the upper limit of the confidence interval for the standard deviation (5% level of significance for a one-tailed test) presented in Figure 6 (which was based on the assumption of normality). Figure 9 shows how many times (*k*) this upper limit (grey line) and the quantile of 95% from bootstrapping (black line) are larger than *s* for each sample size *n*. It can be observed that *k* was lower in bootstrapping than in the normal distribution. This means that the experimental data from TS1 were better than expected from a normal distribution. This difference is more important for small sample sizes and tends to disappear when *n* increases. If we take the sample size suggested in Section 2.2 for the full test procedure, *n* = 24, the bootstrapping results indicate a value of *k* = 1.28, close to and lower than the value of *k* = 1.33 obtained from the normal distribution. Consequently, a smaller sample size could be considered for the full test procedure to reduce the fieldwork. The value of *k* = 1.33 was obtained from bootstrapping when *n* = 18. 

## 4. Conclusions

Currently, a standardised procedure for assessing the centring uncertainty of geodetic and surveying instruments to determine their suitability for the intended and immediate task in field conditions is lacking. Within the framework of the standard ISO 17123, we propose such a procedure through which any user can perform a Type A evaluation, avoiding using values from Type B evaluations which could be unsuitable for the conditions of the intended task. The testing methodology includes a configuration of the test field, measurements and calculation, following the structure of each part of the standard ISO 17123. It can be directly applied to theodolites and total stations. In the case of GNSS receivers, it could be applied if their tribrachs are interchangeable and they incorporate the plummet. The proposal can be applied to an instrument with any type of plummet (laser or optical) not requiring any additional equipment.

The foundation of the method lies in the setting up of stable targets at short distances from the instrument (in the order of 5 m) so that a small displacement in the position of the latter (due to the centring error) leads to a significant variation of the horizontal angle between both targets. That approach is just the opposite of the configuration of the test field in the standard ISO 17123-3, which locates the targets 100–250 m away to minimise the influence of the centring error. A circular standard deviation is obtained as an estimation of the centring uncertainty. Expanded uncertainty can be obtained by applying coverage factors from a circular standard distribution. The procedure also allows detecting the presence of a systematic error in the centring, which is also included in the uncertainty result.

An experimental application was performed with two total stations TS1 and TS2 with different horizontal angle uncertainties ($s_{ISO-THEO-Hz}$ = 15^cc^ and 3^cc^ respectively) and plummet types (laser and optical). In addition, different instrument heights were considered as well as two types of ground mark. The centring uncertainty results are quite different for both instruments. For TS1, a result of 0.25 mm was obtained, which is better than that indicated by the manufacturer. The result for TS2 was quite higher, 0.76 mm, and furthermore the plummet system seems to have been clearly affected by a systematic error.

A set of statistical tests was performed. Results point out that both components (X and Y) of the centring error are independent but they are not conclusive about the normality. The two types of ground mark used did not have any influence on the variance of the centring error, neither did the instrument height. The results also confirmed the lower uncertainty in the centring of TS1 and the presence of a systematic error (bias) in the plummet system of TS2. In addition, a bootstrapping procedure from data of TS1 indicated that the repeatability was better than that expected from a normal distribution. Therefore, the required sample size could be reduced to speed up the fieldwork.

Future research includes the adaptation of the procedure for TLS. In addition, a wide analysis of the influence on the centring uncertainty of different types of ground mark remains an interesting topic.

## Figures and Tables

**Table 1:** Terminology and values in mm suggested by some authors for the centring uncertainty of the instrument (modified from [5]).

Source	Term Used	Type of Centring	Value (mm)
[2]	Max error	Plumb bob	5
		Centring rod	2
		Optical plummet	1
		Forced centring (pillar)	0.2
[13]	Accuracy	Plumb bob	1–2
		Centring rod	1
		Optical plummet	1
[14]	Standard deviation	Plumb bob	1–3
		Optical plummet	0.5–1
		Constrained centring	0.1
		Pillar	≤0.1
[15]	Experimental standard deviation	Plumb bob	1–2
		Optical or laser plummet	0.5
		Centring rod	1
[16]	Accuracy	Plumb bob	3–5
		Centring rod	1
		Optical plummet	0.5
		Forced centring	0.03–0.1
[17,18]	Accuracy at a height of 1.5 m	Laser plummet	1.5
[19]	σ	N/A	0.5–3
[20]	Accuracy	Plumb bob	3–5
		Optical plummet	0.5–1
		Forced centring	0.1–0.3
[10]	Accuracy at a height of 1.55 m	Plummet camera	0.5

**Table 2:** Contribution to the uncertainty of a horizontal angle $s_{\alpha_{CI}}$ due to the uncertainty in instrument centring $s_{CI}$ to targets at distance D and α=100 grad.

$s_{CI}\;(\mathrm{mm})$	D (m)	$s_{\alpha_{CI}}({}^{\mathrm{cc}})$
0.33	5	60
	10	30
	15	20
	20	15
	25	12
0.5	5	90
	10	45
	15	30
	20	23
	25	18
1	5	180
	10	90
	15	60
	20	45
	25	36

**Table 3:** Results from the preliminary test. Variation of the horizontal angles from each position.

Position	Angle	Value (Grad)	Difference	
			(^cc^)	(mm)
A	*α*_A_	100.0527	387	2.2
C	*α*_C_	100.0140		
B	*β*_B_	100.1425	401	2.2
D	*β*_D_	100.1024		

**Table 4:** Maximum value of $s_{ISO-THEO-Hz}$ required for the instrument for different values of $s_{CI}$.

$s_{CI}\;(\mathrm{mm})$	$s_{ISO-THEO-Hz}({}^{\mathrm{cc}})\;(\max.)$
0.1	4
0.25	11
0.5	21
0.75	32
1	42

**Table 5:** Minimum value of $s_{CI}$ which can be assessed for different values of $s_{ISO-THEO-Hz}$.

$s_{ISO-THEO-Hz}({}^{\mathrm{cc}})$	$s_{CI}\;(\mathrm{mm})\;(\min.)$
5	0.12
10	0.24
15	0.35
20	0.47
30	0.71

**Table 6:** Number of setups that have been performed for the experimental application.

Instrument	Ground Mark	Height	Tribrach Turn	No. of Setups
TS1	M1	1.432	A, B, C	3, 3, 3
		1.549	A, B, C	3, 3, 3
		1.673	A, B, C	3, 3, 3
	M2	1.442	A, B, C	3, 3, 3
		1.594	A, B, C	3, 3, 3
		1.684	A, B, C	3, 3, 3
TS2	M1	1.064	A, B, C	3, 3, 3
		1.433	A, B, C	3, 3, 3
		1.654	A, B, C	3, 3, 3
	M2	1.433	A, B, C	3, 3, 3
		1.654	A, B, C	3, 3, 3

**Table 7:** Results (*p*-values) from normality.

Test	TS1		TS2	
	$\;e_{CI-X}\;$	$\;e_{CI-Y}\;$	$\;e_{CI-X}\;$	$\;e_{CI-Y}\;$
Shapiro-Wilks	0.027	0.012	0.026	0.003
Jarque-Bera	0.032	0.130	0.133	0.092

**Table 8:** Results (*p*-values) from Levene’s test for each variable.

Variable	TS1		TS2	
	$\;e_{CI-X}\;$	$\;e_{CI-Y}\;$	$\;e_{CI-X}\;$	$\;e_{CI-Y}\;$
Ground Mark	0.961	0.286	0.386	0.064
Instrument Height	0.634	0.249	0.311	0.045
Tribrach Turn	0.257	0.558	0.333	0.295

**Table 9:** Results (*p*-values) from a global Levene’s test for each instrument.

Variable	$\;e_{CI-X}\;$	$\;e_{CI-Y}\;$
Instrument	<0.001	<0.001

**Table 10:** Results (*p*-values) from an ANOVA.

Variable	TS1		TS2	
	$\;e_{CI-X}\;$	$\;e_{CI-Y}\;$	$\;e_{CI-X}\;$	$\;e_{CI-Y}\;$
Tribrach Turn	0.801	0.381	<0.001	<0.001
