# Correction: García-Balboa, J.L., et al. A Field Procedure for the Assessment of the Centring Uncertainty of Geodetic and Surveying Instruments. *Sensors* 2018, *18*, 3187

**DOI:** 10.3390/s19051036

**Published:** 2019-02-28

**Authors:** José L. García-Balboa, Antonio M. Ruiz-Armenteros, José Rodríguez-Avi, Juan F. Reinoso-Gordo, Juan Robledillo-Román

**Affiliations:** 1 Departamento de Ingeniería Cartográfica, Geodésica y Fotogrametría, Universidad de Jaén, 23071 Jaén, Spain; amruiz@ujaen.es; 2 Centro de Estudios Avanzados en Ciencias de la Tierra (CEACTierra), Universidad de Jaén, 23071 Jaén, Spain; 3 Departamento de Estadística e Investigación Operativa, Universidad de Jaén, 23071 Jaén, Spain; jravi@ujaen.es; 4 Departamento de Expresión Gráfica Arquitectónica y en la Ingeniería, Universidad de Granada, 18071 Granada, Spain; jreinoso@ugr.es; 5 Independent Researcher, 18011 Granada, Spain; jrobledilloroman@gmail.com

The authors wish to make the following corrections to this paper [1]:

## 1. Change in Equations

The authors wish to make the following corrections to Equations (4) and (5):

(4) $$\alpha_{i}=x_{i,2}-x_{i,1},\;\beta_{j}=x_{i,3}-x_{i,2}.$$

(5) $$s_{\alpha }=\frac{\sum_{j=1}^{n}\left(\alpha_{j}-\bar{\alpha }\right)}{n-1},\;s_{\beta }=\frac{\sum_{j=1}^{n}\left(\beta_{j}-\bar{\beta }\right)}{n-1}$$

should be replaced with

(4) $$\alpha_{i}=x_{i,2}-x_{i,1},\;\beta_{i}=x_{i,3}-x_{i,2}.$$

(5) $$s_{\alpha }=\sqrt{\frac{\sum_{i=1}^{n}{\left(\alpha_{i}-\bar{\alpha }\right)}^{2}}{n-1}},\;s_{\beta }=\sqrt{\frac{\sum_{i=1}^{n}{\left(\beta_{i}-\bar{\beta }\right)}^{2}}{n-1}}$$

The authors would like to apologize for any inconvenience caused to the readers by these changes.
